# Germline expression of Imp-L2 in Drosophila females enhances reproductive activity and longevity

**DOI:** 10.1080/19768354.2025.2480150

**Published:** 2025-03-17

**Authors:** Sujin Noh, Sungjoon Na, Xinge Song, Seogang Hyun

**Affiliations:** Department of Life Science, Chung-Ang University, Seoul, Republic of Korea

**Keywords:** Insulin-like growth factor binding protein, Imp-L2, reproductive aging, lifespan, Drosophila

## Abstract

The Imaginal morphogenesis protein-Late 2 (Imp-L2) in *Drosophila* is recognized as a functional homolog of the insulin-like growth factor (IGF) binding protein family. In this study, we report that Imp-L2 expression in germline cells during oogenesis simultaneously enhances both fecundity and lifespan in female *Drosophila*. Loss of Imp-L2, either through knockout or germline-specific knockdown, resulted in decreased reproductive activity, as evidenced by reduced ovary size and fecundity, along with a higher proportion of infertile flies. Conversely, overexpression of Imp-L2 specifically in germline cells enhanced reproductive activity. Imp-L2 appears to regulate germline stem cell proliferation and differentiation independently of IGF signaling. Interestingly, germline-specific knockdown of *Imp-L2* shortened the lifespan of female flies, whereas its overexpression extended it. Thus, Imp-L2 expression in the germline promotes both reproductive activity and longevity, presenting an exception to the typical trade-off between reproduction and lifespan.

## Introduction

The decline in reproductive function with age has significant evolutionary implications, as reproductive success is central to an organism's fitness. While most aging research has focused on somatic decline, comparatively little is known about the factors driving reproductive aging. In humans, the trend of delayed childbearing has led to an increase in individuals experiencing reduced fertility and reproductive capacity with advancing age (Billari et al. 2007). However, reproductive aging is not unique to mammals. In the invertebrate model *Drosophila melanogaster*, egg production decreases in middle age, partly due to reduced germline stem cell (GSC) division and lower survival rates of developing eggs. This decline may stem from factors such as a reduced number of ovarioles, slower GSC division, and increased apoptosis in the egg chambers of older females (Zhao et al. 2008).

Across species, conserved mechanisms may regulate reproductive decline and link reproduction with lifespan. Similarities have been observed in gametogenesis across various organisms, and the regulation of ovulation is conserved between *Drosophila* and humans (Sun and Spradling 2013). The relationship between reproduction and lifespan is a fundamental concept in life-history strategies. Many species exhibit an inverse correlation: high reproductive rates often coincide with shorter lifespans, while longer lifespans are associated with reduced reproductive output. This trade-off reflects the allocation of resources between reproduction and somatic maintenance. However, exceptions to this pattern exist, as some species defy this trade-off by exhibiting both high reproductive output and extended lifespans, influenced by unique ecological or evolutionary factors (Stearns 1992; Kirkwood and Austad 2000; Flatt 2011a; Maklakov and Immler 2016).

In various animals, GSCs must be precisely maintained and proliferated during gametogenesis to ensure successful reproduction (Lehmann 2012; Cheng et al. 2022; Jang et al. 2024). GSCs are the source of gametes, such as sperm and oocytes, and their proper function is essential for reproductive capacity and species continuity. The proliferation and differentiation of GSCs are finely regulated by the interplay of intracellular signaling pathways and the microenvironment. In the ovaries of *Drosophila melanogaster*, GSCs continuously supply gametes through self-renewal and differentiation (Kirilly and Xie 2007; Xie 2008). These processes are governed by extrinsic signals, such as Bone Morphogenetic Protein signaling, as well as intrinsic regulatory mechanisms within the GSCs themselves (Upadhyay et al. 2017). Proper GSC proliferation is crucial for maintaining tissue homeostasis and reproductive potential.

The Imaginal morphogenesis protein-Late 2 (Imp-L2), a homolog of mammalian insulin-like growth factor-binding protein 7, plays a significant role in various developmental processes and regulatory mechanisms in *Drosophila melanogaster* (Honegger et al. 2008; Yun and Hyun 2023). Imp-L2 binds to insulin-like growth factors and inhibits insulin/insulin-like growth factor signaling (IIS), thereby regulating growth and metabolic processes. Interestingly, Imp-L2 has also been shown to promote IIS in specific neurons, contrasting with its systemic role as an IIS antagonist (Bader et al. 2013). These findings suggest that Imp-L2 fulfills diverse roles in developmental and physiological regulation within *Drosophila*.

In this study, we demonstrate that Imp-L2 expressed in germline cells during oogenesis simultaneously enhances both reproductive activity and lifespan in adult female flies. Germline-specific modulation of Imp-L2 consistently affects ovary size, fecundity, fertility and lifespan. Imp-L2 appears to modulate GSC proliferation and differentiation independently of IIS.

## Materials and methods

### Fly rearing

The following fly stocks were obtained from the Bloomington *Drosophila* Stock Center: *w^1118^* (BL 3605), *otu-Gal4* (BL 58424), *UAS-Imp-L2 RNAi* (BL 64936), *Imp-L2 ^Df^* [Df(3L)Excel9000, BL 7921], and *Imp-L2-GFP* (BL 59778). The *Imp-L2 null* fly (*Imp-L2^Def42^*) and *UAS-Imp-L2* have been described previously (Honegger et al. 2008). All stocks were maintained at 25°C with 60% humidity under a 12-hour light/dark cycle on standard glucose-based fly food. The food composition was as follows: 86.2 g anhydrous dextrose, 40.8 g cornmeal, 62.4 g dry yeast, and 9.3 g agar per liter of water. Methyl 4-hydroxybenzoate (Sigma, H3647) and propionic acid (Samchun, P3055) were used as preservatives.

### Measurements of ovary size

Flies were anesthetized on ice and dissected in phosphate-buffered saline (PBS). Ten flies per group were analyzed, and each graph represents the mean of at least five replicates. Ovaries were outlined and their sizes calculated using Toupview software (Touptek). Images of ovaries were captured at 25x magnification using a Zeiss microscope. Statistical analysis was performed using one-way analysis of variance (ANOVA), followed by post-hoc pairwise comparisons with Tukey's Honestly Significant Difference (HSD) test.

### Measurements of fecundity

Female flies were allowed to mate for 3–4 days post-eclosion. Each vial contained one female fly, which was transferred daily to a new vial with fresh food to maintain consistent nutritional conditions. The number of eggs laid by each female was counted daily for approximately two weeks. A minimum of 50 female flies were included in each experiment to ensure adequate sample size. Experiments were performed in triplicate. The infertility rate was calculated by determining the percentage of females that did not lay eggs.

### Lifespan measurement

More than 150 flies were collected three days post-eclosion, with 15 flies placed in each vial. Flies were transferred to fresh food vials every three days, during which the number of dead flies was recorded. This process continued until all flies were deceased. Each procedure constituted one biological replicate, and experiments were repeated at least three times. Lifespan and mortality rate analyses were conducted using the Online Application for the Survival Analysis of Lifespan Assays 2 (OASIS 2) (Han et al. 2016). The Restricted Mean Survival Time (RMST) analysis was performed to compare survival between groups. A two-sample t-test and one-way analysis of variance (ANOVA), followed by post-hoc pairwise comparisons with Tukey's Honestly Significant Difference (HSD) test were performed to evaluate the difference in RMST between groups.

### RNA preparation and quantitative RT–PCR (qRT-PCR)

Fly ovaries were dissected in PBS and homogenized using RNAiso Plus (9109; TaKaRa) for RNA extraction. cDNA synthesis was performed with the BioFACT™ RT-Kit (BR123-10k, BIOFACT). Quantitative PCR was carried out on the CFX Connect Real-Time PCR Detection System (Bio-Rad) using the THUNDERBIRD SYBR qPCR Mix (TOYOBO, QPS-201). Relative mRNA levels were normalized to *Rp49* mRNA and analyzed using the comparative cycle threshold (Ct) method. The primers used were as follows: Imp-L2, 5’- GAG ATC GTT TGC GAG ATG ATG -3’ and 5’- GCT GGC ATA GAT GGT CTT GG -3’; Rp49, 5’- AGG GTA TCG ACA ACA GAG TG -3’ and 5’- CAC CAG GAA CTT CTT GAA TC-3’

### Western blotting

Ovaries from 9-day-old adult females were dissected in PBS and homogenized in lysis buffer. Proteins were separated on 10% polyacrylamide gels and transferred to 0.45 μm Immobilon-P PVDF membranes (Millipore). Membranes were blocked in TBST (Tris-buffered saline, 0.1% Tween 20) with 5% skim milk for 1 h at room temperature (RT). Primary antibodies – anti-phospho-AKT (#4054, Cell Signaling), anti-AKT (#9272, Cell Signaling), or anti-β-actin (#4967, Cell Signaling) – were incubated overnight at 4°C in TBST containing 5% BSA. After washing, membranes were incubated with secondary antibodies in TBST with 5% skim milk for 1 h at RT. Signal detection was performed using a WesternBright ECL kit (K-12045-D20, Advansta) and visualized with a chemiluminescence imaging system (UVITEC).

### Immunohistochemistry

Ovaries from 9-day-old adult females were dissected in PBS and fixed in 4% formaldehyde for 20 min. Tissues were washed three times with PBS containing 0.1% Triton X-100 (PBT) and then blocked in PBT with 5% normal goat serum (NGS) for 1 h at 4°C. Tissues were incubated overnight at 4°C with primary antibodies diluted in the blocking solution: anti-Bam (DSHB 1:20) and anti-Hts 1B1 (DSHB 1:50). After washing, tissues were incubated with secondary antibodies at RT for 2 h. DAPI (1 μg/ml, Molecular Probes) was used for nuclear staining. Images were captured using a Zeiss LSM700 confocal microscope.

### Ovulation analysis

Virgin females were collected within 12 h post-eclosion and maintained on normal food until they were 4–6 days old. To induce ovulation, ten virgin females were mated with fifteen *w^1118^* males in a food vial for 6 h. After mating, females were anesthetized by placing them in a deep freezer for 5 min. The reproductive system of each female was dissected to check for the presence of eggs in one or both oviducts (Sun and Spradling 2013; Lim et al. 2014).

## Results

### Mutation of imp-L2 in the female germline decreases ovary size

**Figure 1.**
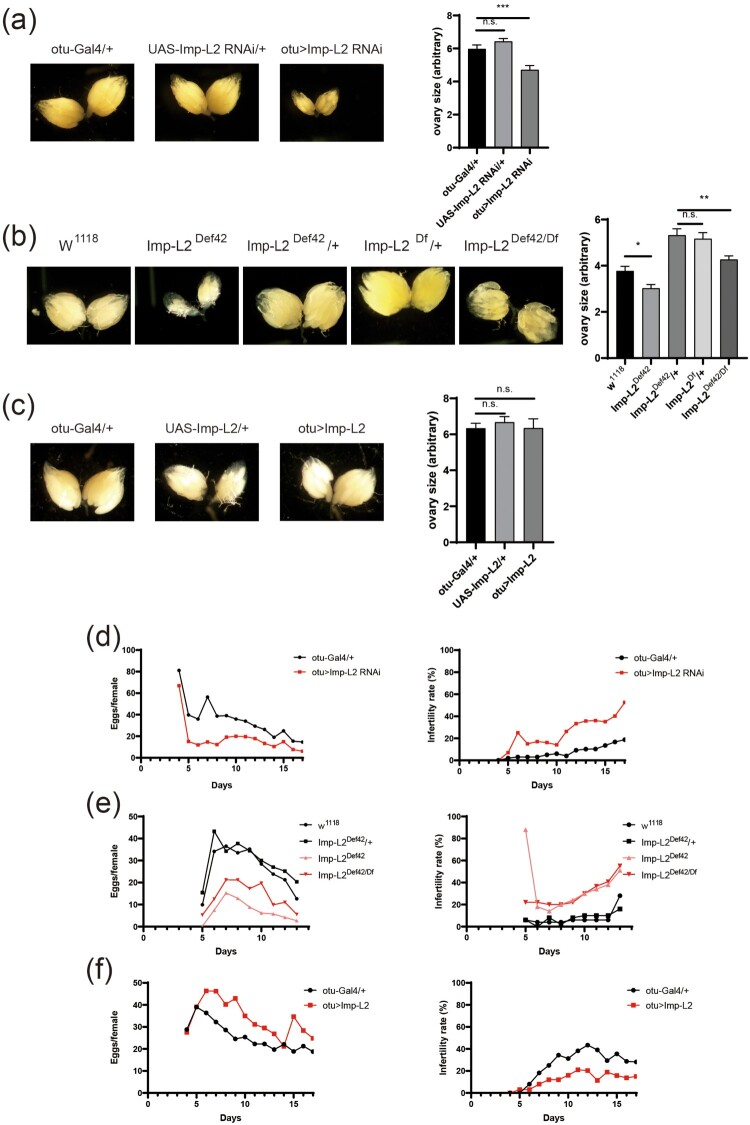
Modulation of Imp-L2 expression affects ovary size and fecundity of female flies. (A) Female germline-specific knockdown of *Imp-L2* using *otu-Gal4* decreased ovary size. One-way ANOVA (F[2, 117] = 15.46; *P* < 0.0001), tukey’s multiple comparisons test; ***P < 0.001. (B) Imp-L2-deficient mutant flies exhibited a significant decrease in ovary size. One-way ANOVA (F[4, 81] = 20.43; *P* < 0.0001), tukey’s multiple comparisons test; *P < 0.05; **P < 0.01. (C) Germline-specific overexpression of Imp-L2 did not significantly affect ovary size. One-way ANOVA (F[2, 70] = 0.1241; *P* = 0.8835), tukey’s multiple comparisons test. (D) Knockdown of *Imp-L2* decreased fecundity (Left) and increased infertility rates (Right). (E) Imp-L2-deficient mutant flies showed reduced fecundity (Left) and increased infertility rates (Right), similar to Imp-L2 knockdown flies. (F) Germline-specific overexpression of Imp-L2 increased fecundity (Left) and reduced infertility rates (Right). n.s.: not statistically significant. Error bars indicate S.E.M. Magnification: 25x.

In *Drosophila*, ovary size serves as a key indicator of reproductive health and fecundity, reflecting the state of GSC proliferation and differentiation. Abnormalities in GSC function or changes in the stem cell niche can affect ovary size, potentially leading to reduced fecundity. External factors such as nutritional status and hormonal signaling also modulate ovary size, influencing reproductive health (Armstrong 2020; Jin and Zhao 2023). Analysis of the expression of Imp-L2 tagged with GFP at native locus (Imp-L2-GFP) (Nagarkar-Jaiswal et al. 2015; Manning et al. 2017) revealed that Imp-L2 is expressed and secreted within the female germarium and GSC niche during oogenesis (Supplementary Figure 1). To examine the impact of Imp-L2 expression on germline cells, we modulated its expression specifically in the germline using the *otu-Gal4* driver, a female germline-specific driver. Knocking down Imp-L2 expression in germline cells resulted in a significant reduction in ovary size (Supplementary Figure 2 and Figure 1A). Similarly, Imp-L2-deficient mutants also showed reduced ovary size (Supplementary Figure 2 and Figure 1B). However, overexpression of Imp-L2 in germline cells did not result in a significant increase in ovary size (Supplementary Figure 2 and Figure 1C). These findings suggest that Imp-L2 expression in germline cells is required for maintaining proper ovary size.

### Imp-L2 expression in the female germline regulates fecundity

Ovary size correlates closely with fecundity, as larger ovaries typically reflect an increased capacity for oocyte production, thereby enhancing reproductive output. Conversely, reduced ovary size is often associated with decreased fecundity. Studies have shown that genetic and nutritional factors influencing ovary size directly impact fecundity, underscoring the importance of ovary size as an indicator of reproductive potential (Garcez et al. 2020; Klepsatel et al. 2020). To determine whether the reduction in ovary size caused by *Imp-L2* mutation affects fecundity, we measured the fecundity of *Imp-L2* mutant female flies. Both germline-specific knockdown and Imp-L2-deficient flies exhibited reduced fecundity and an increased proportion of infertile flies (Figure 1D and 1E). In contrast, increasing Imp-L2 expression in the germline enhanced fecundity and decreased the proportion of infertile flies (Figure 1F). These results confirm that Imp-L2 expression not only influences ovary size but also plays a crucial role in reproductive functionality.

### Imp-L2 expression in the female germline regulates gametogenesis

**Figure 2.**
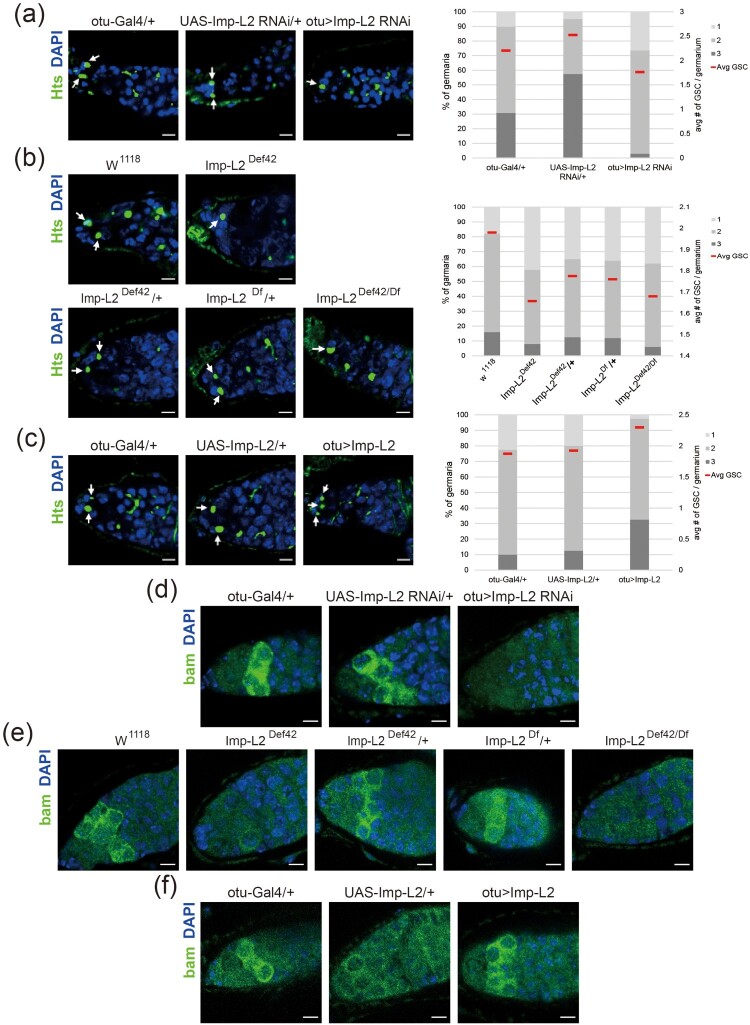
Modulation of Imp-L2 expression affects the number of germline stem cells (GSCs) and subsequent oogenesis. (A, B) Both female germline-specific Imp-L2 knockdown (A) and Imp-L2-deficient (B) flies showed a reduction in the average number of GSCs per germarium, along with an increased proportion of germaria containing fewer GSCs. (C) Germline-specific overexpression of Imp-L2 increased the average number of GSCs per germarium and the proportion of germaria containing a greater number of GSCs. (D) Germline-specific knockdown of *Imp-L2* decreased Bag of marbles (Bam) expression in cystoblasts (CBs). (E) Imp-L2-deficient flies exhibited diminished Bam expression in CBs. (F) Germline-specific overexpression of Imp-L2 increased Bam expression in CBs. Hts (a marker for GSCs) and Bam staining are shown in green, while DAPI staining (nuclei) is shown in blue. Arrows in panels (A – C) indicate GSCs. Scale bar: 5 μm. Magnification: 400x.

In *Drosophila* oogenesis, the proliferation of GSCs and their differentiation into cystoblasts (CBs), the precursor cells of oocytes, are critical processes. Within the ovarian niche, GSCs self-renew and proliferate to maintain a steady supply of cells, while CBs continue development into oocytes, sustaining reproductive capacity (McLaughlin and Bratu 2015). We investigated the effect of Imp-L2 modulation on GSC numbers using Hu-li tai shao (Hts) as a marker for GSC. Hts localizes to the spectrosome, a structure unique to GSCs, and is used to identify GSCs within the niche (Ameku and Niwa 2016). Both Imp-L2 knockdown and mutant flies showed a reduction in GSC numbers, while germline-specific overexpression of Imp-L2 resulted in an increase in GSC numbers (Figure 2A–2C). The expression of Bag of marbles (Bam), a marker for CB differentiation from GSCs, was also affected. Germline-specific knockdown of *Imp-L2* reduced Bam expression, indicating impaired CB division (Figure 2D and 2E) (Mckearin and Spradling 1990; Ji et al. 2017). Conversely, increasing Imp-L2 expression in the germline enhanced Bam expression, suggesting improved CB division (Figure 2F). These results indicate that Imp-L2 positively regulates gametogenesis by promoting both GSC proliferation and CB differentiation during oogenesis.

### Imp-L2 expression in the female germline regulates ovulation

**Figure 3.**
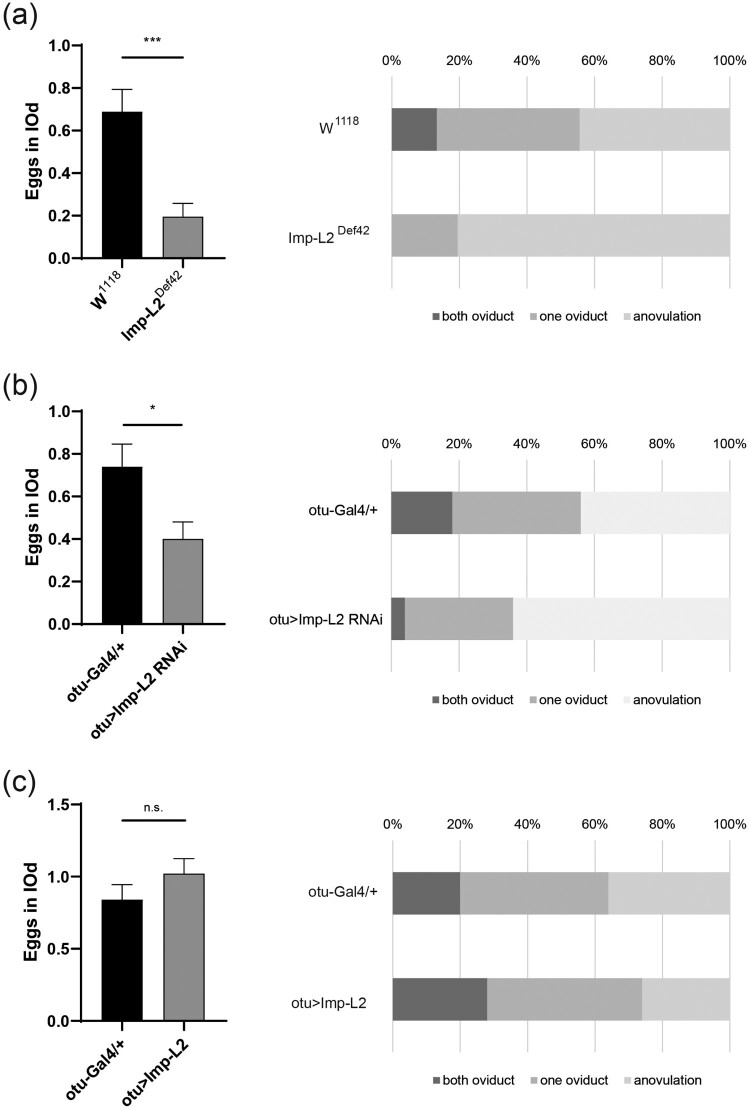
Modulation of Imp-L2 expression affects egg ovulation. (A, B) Both Imp-L2-deficient (A) and germline-specific Imp-L2 knockdown (B) flies exhibited a reduced frequency of ovulation. Ovulation was assessed based on the presence of eggs retained within the lateral oviduct (lOd), with the proportion of flies ovulating from one or both oviducts being determined. (C) Germline-specific overexpression of Imp-L2 showed a slight increasing trend in ovulation frequency, though this was not statistically significant. *p < 0.05; ***p < 0.001 compared to control values (t-test). n.s.: not statistically significant. Error bars indicate S.E.M.

In both Imp-L2 knockdown and mutant flies, the reduced number of GSCs and their impaired differentiation was expected to hinder ovulation. The decrease in GSCs limits the availability of oocytes, and differentiation impairments disrupt the supply of mature oocytes required for regular ovulation. Germline-specific knockdown of *Imp-L2* and Imp-L2-deficient flies exhibited decreased ovulation, as indicated by a reduced presence of eggs in the oviducts after mating (Figure 3A and 3B). Conversely, germline-specific overexpression of Imp-L2 showed a tendency to increase ovulation (Figure 3C). These findings highlight the critical role of Imp-L2 in maintaining GSC function and differentiation, thereby supporting reproductive capacity.

### Imp-L2 expression in the female germline regulates lifespan

**Figure 4.**
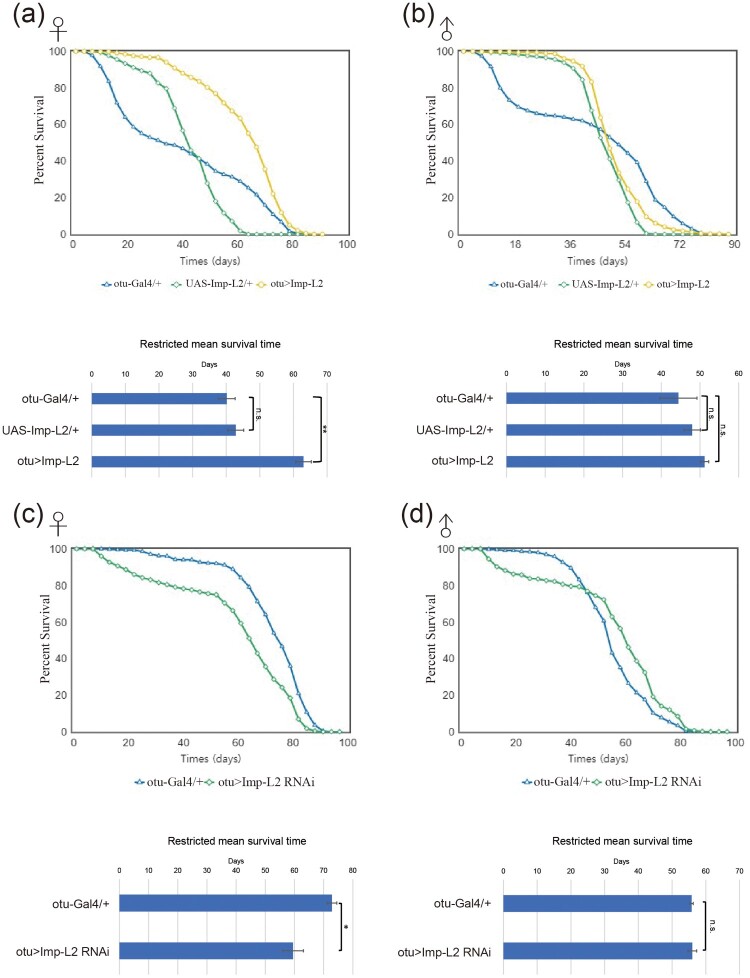
Modulation of Imp-L2 expression affects the lifespan of female flies. (A, B) Germline-specific overexpression of Imp-L2 significantly extended the lifespan of female flies (green) compared to control flies (blue) (A), whereas no effect was observed in males (B). One-way ANOVA (F[2, 6] = 27.31; *P* = 0.001), tukey’s multiple comparisons test; **P < 0.001 (A). One-way ANOVA (F[2, 6] = 1.194; *P* = 0.3659), tukey’s multiple comparisons test (B). (C, D) Germline-specific knockdown of *Imp-L2* reduced the lifespan of female flies (green) compared to controls (blue) (C), with no effect observed in males (D). *p < 0.05 compared to control values (t-test). Note that flies in panels (A) and (B), but not in (C) and (D), carried one copy of the *yw* gene, which appears to negatively affect lifespan. A total of n = 450 flies were analyzed across all groups. Survival rates were assessed in groups of 150 individuals, and the data represent the results from three independent replicates. n.s.: not statistically significant. Error bars indicate S.E.M.

Reproductive activity often correlates inversely with lifespan, with females that produce more offspring frequently showing shortened lifespans (Flatt 2011a; Maklakov and Immler 2016). To determine whether this trade-off occurs with Imp-L2 modulation, we examined the lifespan of female flies. Interestingly, germline-specific overexpression of Imp-L2 not only increased fecundity but also significantly extended the lifespan of female flies (Figure 4A). This effect was specific to females, as male lifespan remained unaffected (Figure 4B). Conversely, germline-specific knockdown of *Imp-L2* reduced the lifespan of female flies but did not affect males (Figure 4C and 4D). These findings demonstrate that Imp-L2 expression in the female germline enhances both reproductive activity and host longevity.

### The observed effects of Imp-L2 expression in the female germline appear IIS-Independent

Since Imp-L2 is known as an IIS antagonist, we examined IIS activity in ovarian tissues following germline-specific modulation of Imp-L2. Unexpectedly, neither knockdown nor overexpression of Imp-L2 in the germline significantly altered IIS activity, as determined by monitoring the active form of Akt (p-Akt) (Supplementary Figure 3). These findings suggest that the observed effects on reproduction and lifespan are independent of IIS activity, warranting further investigation into the underlying cellular mechanisms.

## Discussion

Our findings reveal a previously uncharacterized role of Imp-L2 in the *Drosophila* germline. Prior studies have shown that increased IIS positively regulates ovary size and GSC proliferation, emphasizing its critical role in coordinating nutrient availability with reproductive output in *Drosophila* (Mendes and Mirth 2016; Jin and Zhao 2023). Surprisingly, despite Imp-L2's established function as an IIS antagonist, loss of Imp-L2 resulted in decreased ovary size and GSC numbers without affecting IIS activity in the ovary. This suggests that the phenotypes associated with germline-specific Imp-L2 expression are mediated through mechanisms independent of IIS.

Typically, lifespan and reproduction are considered to exist in a trade-off relationship, where increased investment in one often compromises the other (Flatt 2011b; Takeshita 2024). However, our results indicate that Imp-L2 plays a unique role, simultaneously enhancing both lifespan and reproductive output. This dual effect positions Imp-L2 as a critical regulator at the intersection of longevity and reproduction, challenging the conventional understanding of life-history trade-offs. These findings highlight Imp-L2 as a promising candidate for future research on reproductive aging and infertility. Further investigations are necessary to elucidate the underlying molecular and cellular mechanisms driving these phenotypes, which may offer insights into conserved pathways linking reproduction and lifespan across species.

## Supplementary Material

Supplemental Material

## Fundings

This research was supported by the Basic Science Research Program through the National Research Foundation of Korea (NRF), funded by the Ministry of Education, Science, and Technology (Grant Number: 2023R1A2C1005908). This research was supported by the Chung-Ang University Young Scientist Scholarship in 2023.
