# Supplementary figures and images for: Germline expression of Imp-L2 in Drosophila females enhances reproductive activity and longevity
Source: Anim Cells Syst (Seoul). 2025 Mar 17;29(1):31–40. doi: 10.1080/19768354.2025.2480150 (PMC11915738; doi:10.1080/19768354.2025.2480150)

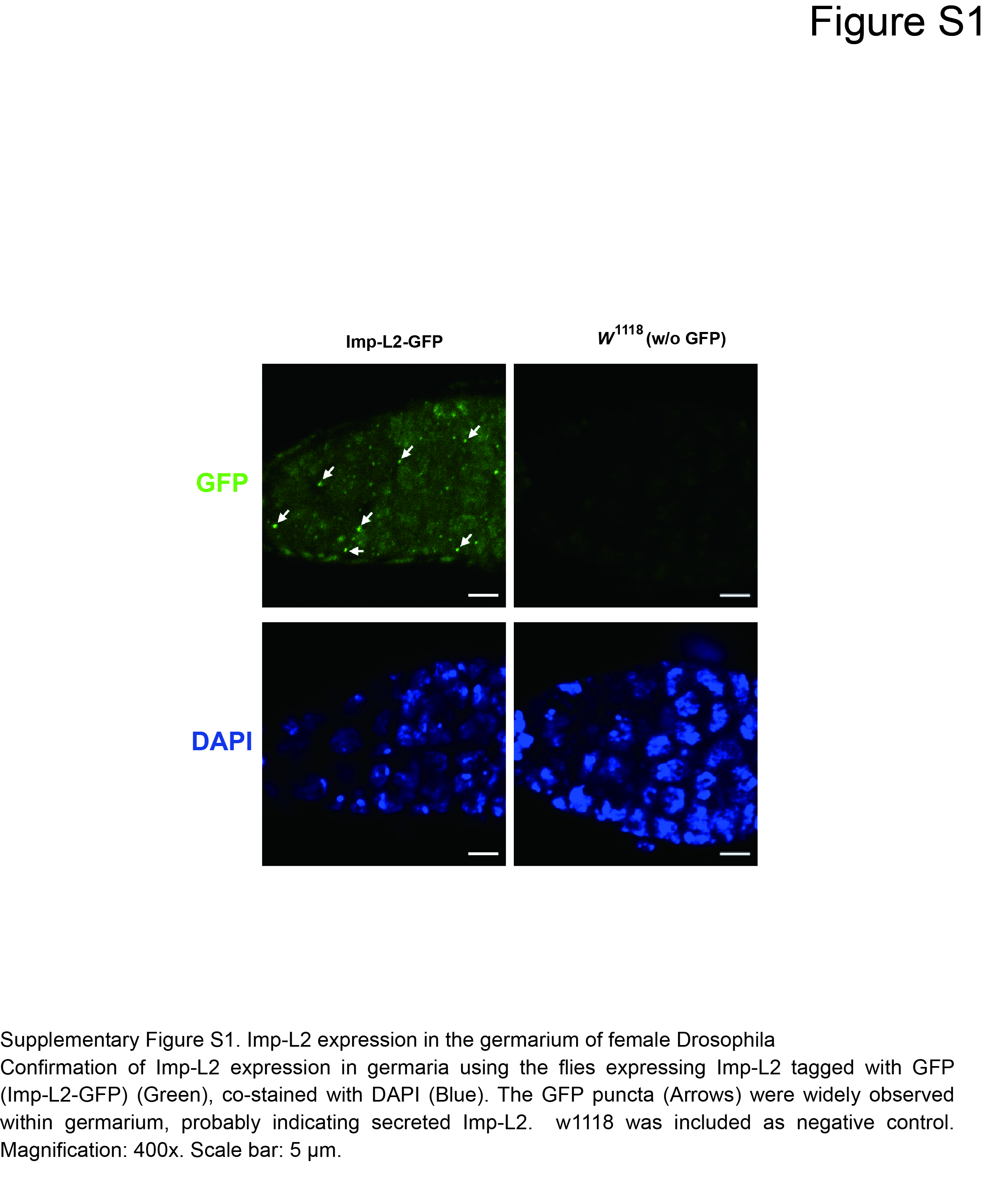

Supplement: Supplemental Material [file TACS_A_2480150_SM9035.zip › s1.jpg]

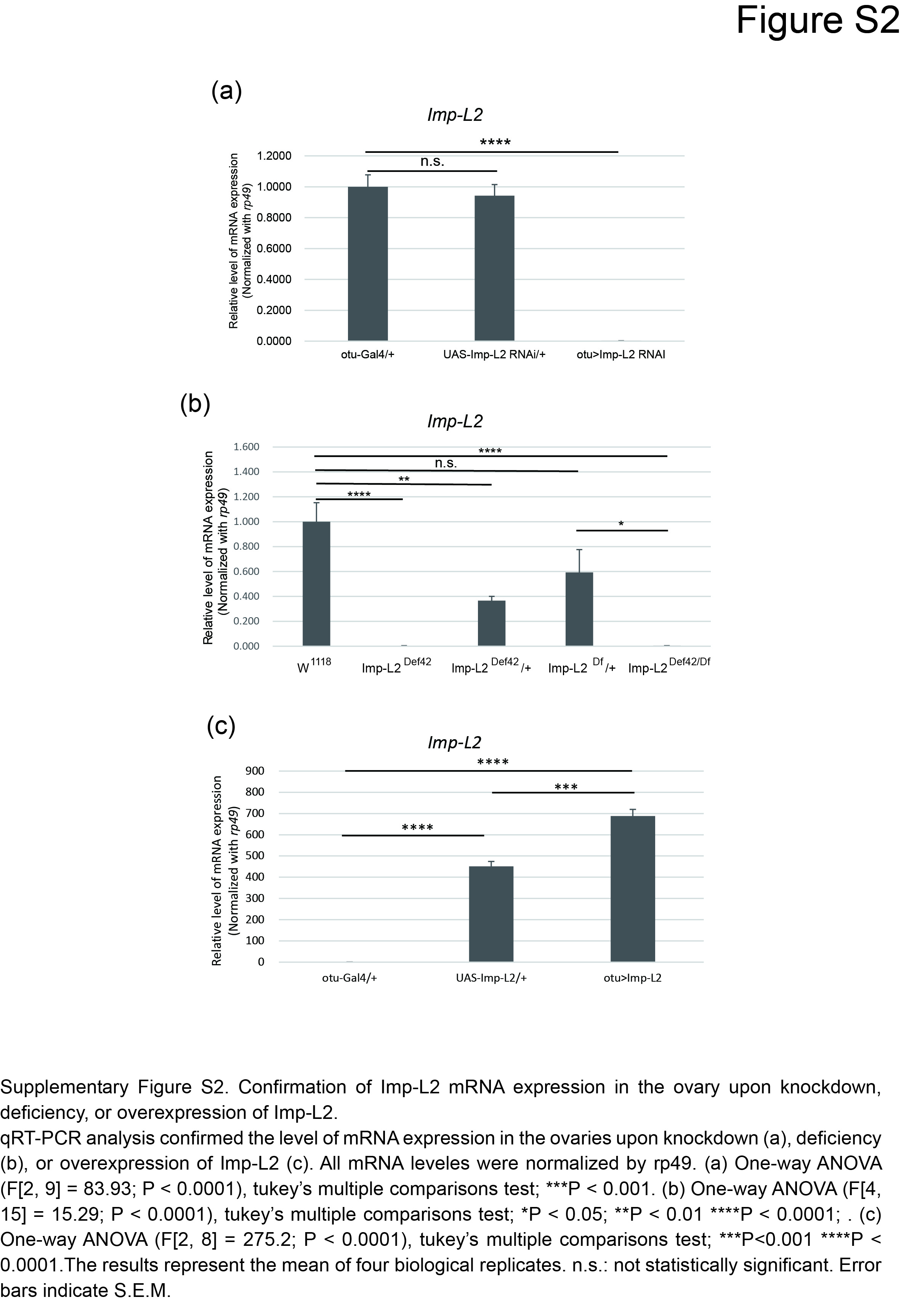

Supplement: Supplemental Material [file TACS_A_2480150_SM9035.zip › s2.jpg]

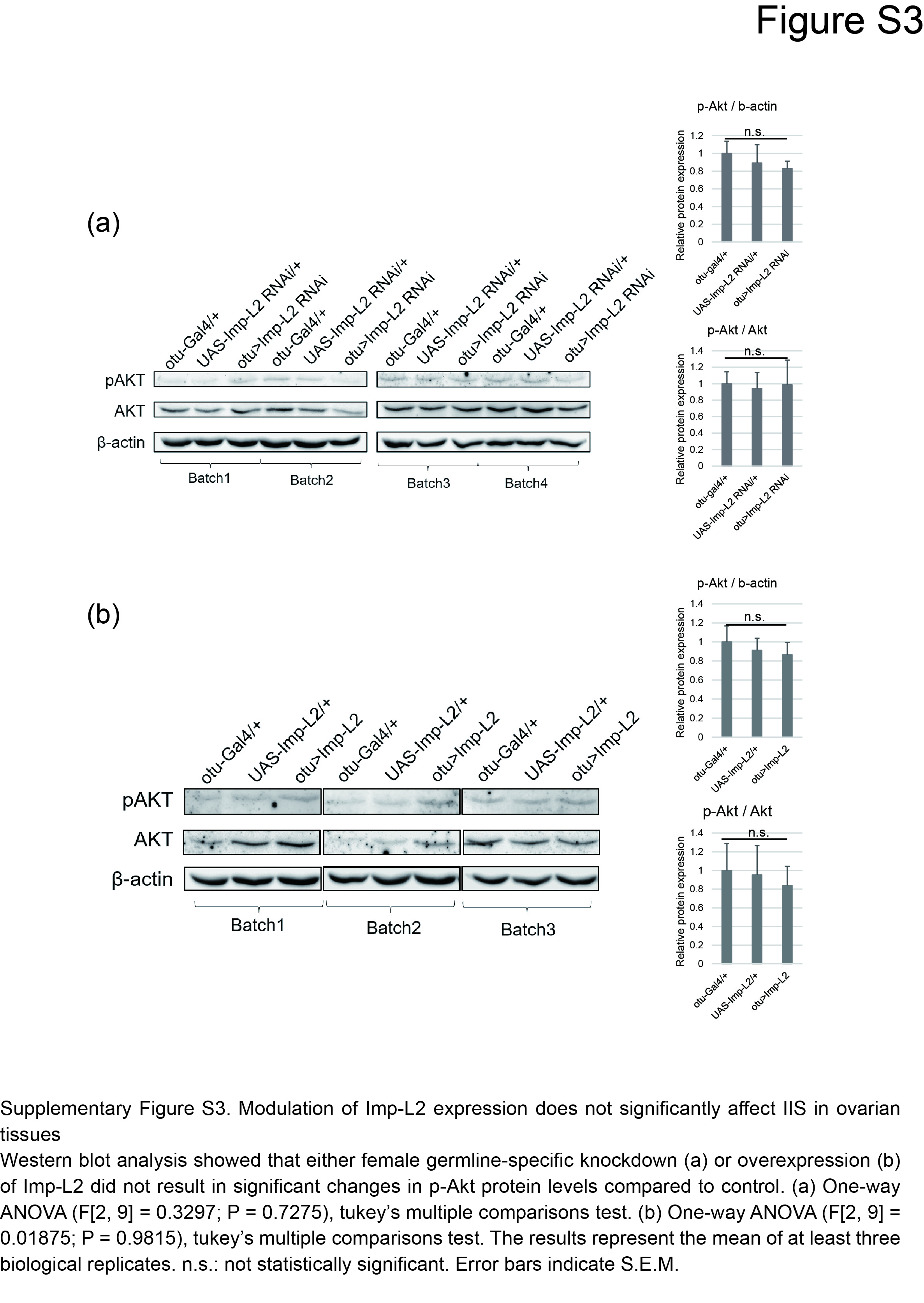

Supplement: Supplemental Material [file TACS_A_2480150_SM9035.zip › s3.jpg]
